# Using ADAPT-ITT to Modify a Telephone-Based HIV Prevention Intervention for SMS Delivery: Formative Study

**DOI:** 10.2196/22485

**Published:** 2020-10-06

**Authors:** Teaniese Davis, Ralph Joseph DiClemente, Michael Prietula

**Affiliations:** 1 Center for Research and Evaluation Kaiser Permanente Georgia Atlanta, GA United States; 2 Department of Social & Behavioral Sciences School of Global Public Health New York University New York, NY United States; 3 Goizueta Business School & Hubert Department of Global Health Emory University Atlanta, GA United States

**Keywords:** short message service, HIV, African Americans, adolescent, female, texting, mHealth, ADAPT-ITT framework, intervention study, health status disparities, young adult, risk reduction behavior

## Abstract

**Background:**

African American adolescent females are disproportionately affected by sexually transmitted infections (STIs) and HIV. Given the elevated risk of STIs and HIV in African American women, there is an urgent need to identify innovative strategies to enhance the adoption and maintenance of STI and HIV preventive behaviors. Texting is a promising technology for creating preventive maintenance interventions (PMIs) that extend the efficacy of the original intervention. However, little guidance in public health literature is available for developing this type of application.

**Objective:**

This paper describes a formative pilot study that incorporates user experience methods to design and test PMI texts for Afiya, an original evidence-based intervention (EBI) specifically designed for African American adolescent females. This study aims to describe the adaptation process of health educator–led phone calling to text-based communication.

**Methods:**

The formative process followed the assessment, decision, adaptation, production, topical experts-integration, training, testing (ADAPT-ITT) framework for adapting EBIs and using them in a new setting, for a new target population or a modified intervention strategy. This study presents the details of how the phases of the ADAPT-ITT framework were applied to the design of the adaptation. An advisory board was constituted from the target population, consisting of 6 African American women aged 18-24 years, participating in formative activities for 12 weeks, and involving components of the PMI design. As Afiya included a telephone-based PMI, developers of the original Afiya phone scripts crafted the initial design of the SMS-based texts and texting protocol. The advisory board participated in the 1-day Afiya workshop, followed by 4 weeks of texting PMI messages and a midcourse focus group, followed by 4 more weeks of texting PMI messages, ultimately ending with a final focus group. At the advisory board’s request, this phase included an optional, additional week of text-based PMI messages.

**Results:**

The methods provided a rich source of data and insights into the fundamental issues involved when constructing SMS-based PMI for this target population and for this EBI. Prior contact and context are essential as the health educator was identified as a key persona in the process and the messages were situated in the original (workshop) context. Narrative adaptations for personas emerged from advisory board discussions. Suggestions on how to expand the PMI to current, specific social contexts indicated that the use of narrative analysis is warranted.

**Conclusions:**

The use of existing EBIs incorporating telephone-based PMI scripts facilitated the initial design of the texts, with a subsequent narrative analysis of the advisory board data providing additional adjustments given the actual context. Additional examination of the advisory board feedback revealed that personas would offer insight into and opportunities for a persona-specific modification of texting narratives.

## Introduction

Young people in the United States have never known a world without HIV [1]. The National HIV and AIDS Strategy has consistently identified African Americans as a population at increased risk for HIV to which prevention efforts and resources should be directed (President Donald Trump closed the Office of National AIDS Policy in 2017) [2]. In recent Centers for Disease Control and Prevention (CDC) reports [3,4], in 2018, African Americans comprised approximately 12.3% of the US population but accounted for approximately 42% of the new HIV diagnoses and a disproportionate rate of HIV deaths at 28.4 per 100,000 population (vs 4.9 per 100,000 for Whites), leading all other racial and ethnic groups. African American females comprised approximately 13% of females in the United States but accounted for 58% of new HIV diagnoses among females, with 92% of infections attributed to heterosexual contact. The infection rate for African American females (21.3 per 100,000 population) was 13 times the rate for White females (1.7 per 100,000). These data underscore the HIV disparities disproportionately burdening African American women.

Disparities persist among adolescents as well. In 2018, the HIV diagnosis rate among adolescents in the United States was highest among African Americans (26.0 per 100,000), which was 19 times the rate for White adolescents (1.4 per 100,000) [5]. Young people aged 13 to 24 years accounted for 21% of new HIV infections, of which 52% were African American. The incidence among persons aged 13 years between 2014 and 2018 revealed that the rate among African Americans was still excessive, being 8 times that of Whites [6]. These disparities highlight a need for increased, effective efforts to address HIV prevention efforts among young African American women.

Challenges to HIV prevention include low testing rates, youth unsure of their HIV status, inadequate or delayed sex education, incorrect or no condom use, substance use, multiple partners, and high sexually transmitted disease rates. The 2017 Youth Risk Behavioral Surveillance Survey results from 144 high schools [7] estimated that African American adolescents, compared with White adolescents, were more likely (15.2% to 7.9%) to have been tested for HIV, more likely to have multiple partners (11.7% to 8.6%), and more likely to have had sex before the age of 13 years (7.5% to 2.1%). The required health education varied widely, especially within large, urban school districts where, across grades 6 through 12, the range was 0% to 100% for each grade (median: 6th grade, 59.9%; 7th grade, 47.6%; 8th grade, 27.0%; 9th grade, 75.9%; 10th grade, 44.4%; 11th grade, 39.1%; and 12th grade, 53.6%) [8]. Even when delivered in those districts, variances were found on coverage of all 20 topics in grades 6 through 8 (range 17.6% to 41.0%) and grades 9 through 12 (range 42.8% to 75.0%) [8].

A systematic review and meta-analysis of extant US school-based HIV and STI prevention programs found *no persuasive evidence for the effectiveness of school-based programs* [9]. However, a recent systematic review specifically examining the impacts on African American adolescents found that *sexual health interventions are associated with increases in abstinence and condom use and improved sexual health knowledge, self-efficacy, and intentions* [10]. It is essential that the response to HIV and AIDS effectively addresses African American youth.

Early prevention programs addressing behavioral risk factors are important for reducing the HIV disparity observed among African American adolescents relative to White adolescents. Although less is known about the burden and epidemiological details of sexually transmitted infections (STIs), especially HIV and AIDS, among youth and adolescents than about infants and adults [11], there is accumulating evidence that designing interventions for adolescents requires unique combinations and insight into gender, developmental level, race, culture, and social factors [12-14]. This is especially salient in the escalated concentration of HIV and AIDS in southern United States [3,15-17]. Understanding these factors in the context of adolescents is essential for designing and delivering effective prevention programs to ensure that they are developmentally appropriate [13,18]. In addition, there is one particularly important question that is salient to this paper: how can the effect of a successful intervention be maintained?

### Program Maintenance Interventions

A review of national STI and HIV prevention programs targeting African American adolescents indicated that the *frequency and duration* of a program’s implementation was related to its efficacy [19]. The implementation fidelity of extended face-to-face programs is difficult to maintain and expensive, particularly in an era of uncertain funding and support for HIV and STI prevention programs [20-23]. A recent metareview of youth-focused interventions concluded that they effectively influenced condom use, sexual health knowledge, and safer sex norms but suggested that future work *should focus on intervention adaptations and supplements that may extend their protective effects over time* [24]. Thus, the problem becomes one of sustaining program effectiveness over time—program maintenance interventions. This echoes the call from a focused review of HIV prevention interventions targeting African American women where technology can serve as *boosters* sustaining the effects of the intervention [25].

In general, preventive maintenance intervention (PMI) strategies such as making phone calls to participants have been more effective in sustaining intervention effects over time compared with interventions without a maintenance component [26]. However, incorporating intervention components to enhance behavior maintenance such as face-to-face programs and phone calls, is difficult to sustain, labor intensive, and costly [23]. Consequently, sexual health researchers have called for the increased use of technology-based interventions to promote the maintenance of HIV and STI preventive behaviors [27,28]. One of the most promising technological platforms is the mobile (smart) phone delivering health-related services—texting.

### The Social Role of Texting

Mobile health (mHealth) technology is gaining recognition in the field because it affords a wide array of user-facing functionality and services [29]. Evidence supporting the feasibility, acceptability, and efficacy of digital interventions is growing [30-32], particularly for adolescents [33-39]. SMS texting is seen as an mHealth technology with specific promise for adolescents [40], thus serving as an opportunistic technology for impacting sexual health behavior [41-45]. For younger adolescents (aged 13-17 years), texting is the preferred mode of communication with friends, surpassing social media apps, face-to-face interactions, and telephone calls [46], with 95% of adolescents (African Americans: 94%) having access to a smartphone and 89% of adolescents being on the web *almost constantly* or *several times a day* [47].

The critical point is that texting is a social medium. As opposed to theoretical stances that presume the *socialness* of technology is heavily determined by the characteristics of the channel’s ability to provide cues, we adopted a social information processing perspective. This suggests that individuals adapt available cues for a given channel (eg, language, textual display options) to successfully accommodate their management of social interactions [48,49] over time. Technological characteristics are less about limiting the *amount* of social information exchanged and more about determining the *rate* of that exchange [49]. Furthermore, the social information processing approach is consistent with co-construction theories of adolescent development and technology-mediated exchanges, wherein adolescents are *cocreating the internet environment through processes of social interaction* and *construct the same developmental issues online as they do off* [50]. It is about establishing a common conceptual framework shared between the health educator and the client.

Adolescents’ online and offline worlds are strongly connected as *they use online communication for offline issues, and to connect with people in their offline lives* [51]. Indeed, research on Twitter and Facebook has shown remarkable structural similarities between online social networks and offline (face-to-face) networks [52]. This could include direct mapping between the online and offline networks as well as the inclusion of online-only individuals, thus treating the modes of communication as *essentially the same* [52]. This explains the explosion of messaging among adolescents for relationship management. For adolescents, texting is as much a social choice as a technical choice. In fact, it is often the preferred choice [53], surpassing social media apps and phone calls, with adolescent females more likely to use texting as conduits for conversations with friends [54].

Consequently, we suggest that interventions beginning by establishing a strong social context (eg, initial meetings, workshops) would benefit from a texting-based PMI that can retain the social presence of that context by affording social presence cues to enhance the message [55]. The belief is that such hybrid intervention models can help sustain the impacts of EBI by sustaining the narrative engagement of participants. A mechanism we are developing to sustain narrative engagement is the tailoring of PMI messages via the specification of context-determined personas.

As with other intervention components, PMIs must be carefully designed and implemented, otherwise they may undermine the initial intervention [23]. Reviews of texting or mobile apps intended to increase various specific preventive or adherence behaviors in adolescents (eg, sexual health, smoking, oral health, sickle cell disease) reported that the overall efficacy findings were modest, but again only a minority reported a theoretical framework in their design [34]. The feasibility and accessibility of SMS as a behavioral maintenance strategy for African American adolescent populations with increased risk of HIV and STI acquisition requires *continued adaptation of evidence-based interventions with text messaging–enhanced content to expand our knowledge of the potential of this approach* [56].

Therefore, instead of starting with the technology, start with a theoretically grounded EBI, then discern how to *adapt* the EBI to technology to each other. The integrity of the EBI must be maintained whereas the capability of the technology must be exploited in the context of the target population. Although a variety of *generic* approaches (design guides or review recommendations) for integrating technology into behavioral change interventions have been proposed [57-62], a more focused approach was appropriate for this adaptation. We selected a design framework to guide how the *intervention can be* *systematically* *adapted to a technological form and use*.

In this paper, we present a formative pilot study that describes the design process applied to adapting an EBI to reduce the risk of HIV and STI among African American adolescent females. We selected an existing EBI for this target group that would likely benefit from a text-based program maintenance intervention to extend the EBI’s reach, sustainability, and effectiveness. We applied an adaptation-implementation framework that demonstrated efficacy for that purpose—the Assessment, Decision, Adaptation, Production, Topical experts—Integration, Training, Testing (ADAPT-ITT) framework [63].

### ADAPT-ITT Framework and Implementation Science

Calls to advance implementation science argue for the development of adaptation strategies *that would more comprehensively describe the needed fit between* *interventions and their settings* [64]. A recent review [65] traces the history of the most prominent models, including the ADAPT-ITT framework, noting the primary health context of their application and extracts steps that is common across frameworks. These frameworks reflect the CDC’s earlier general guidance describing the ADAPT process intended to assist health departments and community-based organizations in adapting an EBI *to fit the cultural context, risk determinants, risk behaviors, and unique circumstances of the agency without competing with or contradicting the core elements and internal logic* [66].

The purpose of this research is to use the ADAPT-ITT framework to modify existing EBIs to increase STI and HIV preventive behaviors among African American females. Adaptation focuses on translating health educator–facilitated phone calls to SMS-delivered messages. We selected the ADAPT-ITT framework because of our experience with the framework, its relevance to our target population, and its demonstrated effectiveness in guiding the design implementation of postworkshop PMIs to extend elements of the primary intervention over time [67]. The ADAPT-ITT framework is being widely used to design and implement HIV and STI prevention interventions and other prevention adaptations in diverse settings in different cultural contexts, both domestically and internationally [68-78].

## Methods

This was a formative study using qualitative methods. ADAPT-ITT has 8 phases. Each phase brings contextual nuances and constraints that determine how the phases are engaged and the timing of corresponding tasks. The phases are as follows: (1) Assess the proposed new priority population’s HIV risk profile, (2) Decide on whether to adopt or adapt an EBI, (3) Administer novel methods to facilitate the adaptation process, (4) Plan on what aspects of the EBI need to be adapted and plan on how best to evaluate the adapted EBI, (5) identify Topic experts to assist in the adaptation process, (6) Integrate material from the topic experts to adapt the EBI, (7) Train staff to implement the adapted EBI, and (8) Test the adapted EBI. Our texting adaptation process describes how the ADAPT-ITT phases can be used to guide the adaptation of EBI components to texting.

The goal was to select an existing EBI for African American adolescent females who would benefit from text-based PMIs to extend the EBI’s reach, sustainability, and effectiveness. We applied the ADAPT-ITT framework to translate an original EBI (Afiya) that included a face-to-face workshop followed by health educator phone call PMIs to an adapted intervention that would include the same face-to-face workshop but followed-up by health educator texting PMIs. Table 1 summarizes the 8 steps and the lessons learned in the context of this adaptation pilot. The lessons learned identified as *structure* describe changes made to EBI delivery or logistics. The lessons learned identified as *content* describe changes to the information delivered in the intervention. The core element of the translation process is the formation of an advisory board (advisory board) selected from the target population.

**Table 1:** Application of the Assessment, Decision, Adaptation, Production, Topical experts—Integration, Training, Testing framework with a preventive maintenance intervention texting adaptation in Afiya.

Phase			Methodology decisions		Results or observations
1. Assessment			Formed advisory board Conducted an initial meeting with advisory board Conducted meetings with intervention experts for the target population		The target population (African American adolescent females) requires interventions addressing their specific situation The intervention needs to address safer sex norms, sexual negotiation, and refusal skills, HIV and STI^a^ preventive attitudes The advisory board was selected for project, met, and provided input on the plan Advisory board members were paid as consultants to develop or test the feasibility of texting PMI^b^
2. Decision			Selected HORIZONS and Afiya interventions Decided to adapt Afiya phone-based PMIs to texting PMIs Original developer of Afiya-led adaptation mHealth^c^ and texting literature reviewed		HORIZONS, an evidence-based intervention that successfully reduced STIs and increased condom use among young women, was selected as it appropriately addressed the target population Afiya selected as it demonstrated a PMI based on HORIZONS to the same target population, but extend the efficacy of the HORIZONS intervention Texting is most likely the simplest mHealth adaptation to adapt from Afiya and appropriate implementation technology choice for the target population
3. Administration			Decided not to modify Afiya core elements Decided to focus on Afiya core element support by texts Adaptation would involve scripts and texts responses based on Afiya documents and data		Retention of primary intervention form assured standard of care and served as a shared primary intervention control (therefore, theater testing was not conducted) 5 core elements of Afiya were identified to be supported by PMIs
4. Production			Designed 8-week iterative formative pilot plan for adaptation Designed questionnaires and focus group–led questions Designed initial texts and support scripts Selected company for formative pilot texting Reviewed with Afiya and HORIZONS creators Reviewed plan with advisory board Assembled Afiya workshop materials		Iterative formative pilot designed for advisory board Primary intervention led by Afiya and HORIZONS developers in formative pilot and support scripts and associated text messages were created Important to have both advisory board and intervention developers review pilot design and timing Important to have intervention developers review PMI scripts and texting form or content (Draft 1 completed) Texting platforms vary in terms of capacity, functionality, and cost. Total program costs should be estimated
5. Topical experts			Obtained review advice regarding HIPAA^d^ issues relevant to texting Obtained review advice regarding pre- and postintervention CASI^e^ instruments for future effectiveness pilot Met with texting companies (with advice from IT^f^) for future effectiveness pilot		CASI content should be reviewed for duplication or replication as well as total time required (test run the instrument) Texting is not encrypted, so both HIPAA (and local IRB^g^/state) requirements must be reviewed regarding texted data and data privacy issues Role, functionality, and cost of texting companies were more clearly defined with this experience Overall costs for the 8-week formative pilot were made
6. Integration			Integrated Draft 1 and topical expert comments for scripts, SMS texting content or form (Draft 2) Conducted Afiya workshop with advisory board Integrated topical expert comments of pre- and postworkshop CASI materials Integrated Draft 2 and CASI components (Draft 3) Final review by advisory board and Afiya and HORIZONS creators		Draft 2 completed Draft 3 completed Final formative pilot design completed
7. Training			Created training materials for the formative pilot		Formative pilot materials completed
**8. Testing**			Submitted to IRB for clearance of the formative pilot		Obtained IRB clearance to determine adaptation efficacy
	*Step 1: formative pilot*				
		Conducted 8-week formative pilot with advisory board: primary Afiya intervention, 4-week texting, focus group 1 (adjustments), 4-week texting, focus group 2 (adjustments)		Iterative pilot design provided important feedback on texts (timing, wording, dose) as well as script and CASI modifications Information gathered during the first advisory board focus group was applied to modifying the second 4-week texting session, information gathered during the second 4-week focus group was also useful (topic experts were consulted during these sessions as necessary) 5 personas identified, exit interviews added Results informed design of adaptation impact pilot	
	*Step 2: feasibility pilot*				
		Designed texting PMI RCT^h^ feasibility pilot study Study methods submitted to IRB for approval		Obtained IRB clearance for 3-arm feasibility pilot study Preparing for PMI RCT feasibility pilot study to examine short-term intervention efficacy	

^a^STI: sexually transmitted infection.

^b^PMI: preventive maintenance intervention.

^c^mHealth: mobile health.

^d^HIPAA: Health Insurance Portability and Accountability Act.

^e^CASI: computer-assisted self-interview.

^f^IT: information technology.

^g^IRB: Institutional Review Board.

^h^RCT: randomized controlled trial.

### Phase 1: Assessment

*Who will be the primary audience for the EBI?* Phase 1 included incorporating information from the published literature on the need for behavioral maintenance strategies that are sustainable, encouraging health behavior maintenance (as previously summarized). The epidemiological data for this target population highlighted the health disparities in the prevalence of HIV transmission through heterosexual contact among African American female adolescents and young women. Our target population was well defined, and the population attributes and risk profiles were already matched with EBI theory and design approaches to interventions. Therefore, we could immediately recruit an advisory board from that target population. The advisory board members consisted of 6 African American women aged 18-24 years to serve as additional assessment-informative roles throughout the adaptation process. The advisory board members were compensated to participate in the formative advisory activities for 12 weeks.

### Phase 2: Decision

#### What EBI Will Be Used and Will It Be Adapted?

In phase 2, the critical decision on which EBI to select or to determine whether any EBI would be appropriate is made. This necessitates an examination of EBIs and of how likely the target population is to respond to the adapted components of the intervention. In this case, texting. This provides the theoretical and empirical foundation for the form and substance of the texting PMI.

#### Selection of the Primary EBI: Afiya

We examined several HIV and STI interventions explicitly designed and tested for either Blacks, Black females, Black adolescents, or Black adolescent females. Afiya was selected as the intervention to be adapted for the project for 3 reasons. First, Afiya [79] incorporates the HORIZONS intervention that has been evaluated and designated a tier I (best) evidence-based risk reduction intervention by the CDC [80]. HORIZONS, an in-person group-delivered session tailored for Black adolescent females, significantly reduced chlamydial infections and increased HIV and STI preventive behaviors, partner communication efficacy, condom use efficacy, and HIV and STI prevention knowledge. Importantly, the underlying theories of HORIZONS and Afiya are social cognitive theory [81] and the theory of gender and power (TGP) [82-84]. The TGP addresses partner influences and gender-based social correlates that influence behavioral risk factors, which have been extended to address the exposures, social or behavioral risk factors, and biological properties that increase women’s vulnerability to HIV acquisition [83,85].

Second, Afiya used HORIZONS plus bimonthly phone calls for 3 years to determine whether healthy behaviors could be maintained over time. The calls were brief, tailored one-on-one sessions with a health educator, delivered over a 36-month period, and the calls served as PMIs. In the Afiya trial [79], the workshop (tested in HORIZONS) was implemented as a single 4-hour group session delivered by trained African American female health educators. When answering a call, health educators followed an algorithm in their call scripts to guide them through scenarios involving intimate and sexual partnerships, referencing information from the workshop. Afiya (1 workshop+18 phone calls) was efficacious. Over the 36-month follow-up, compared with the control group, participants in the treatment group had significantly lower incidences of chlamydial (50% reduction) and gonococcal infections (60% reduction), a higher proportion of condom use (both within 90 days and 6 months before assessment), and fewer sexual episodes when high on drugs and/or alcohol [79]. These findings provide initial insights into (1) how to begin to adapt the telephone-based PMI to an SMS-based PMI and link them to core elements of the intervention theory and (2) how the target population would respond to a communication mode that differed from the initial telephone calls.

Third, the developers of both HORIZONS and Afiya were available for consultation and reviewed the project products to maintain the integrity of the adaptation and ensure that it remained faithful to the theoretical components of the original intervention.

#### Selection of Texting Adaptation

As discussed, simple mobile phone–based texting was selected as it is ubiquitous, tethered to individuals, and a widely accepted technological application, especially in the target population (teen or adolescent African American women). Using mobile phone technology for adolescent and young adult health purposes was promising as most members of this population possess this technology and exhibit distinct patterns of use, especially the enduring dominance of texting [40,86,87]. Thus, in terms of reach, texting affords a *preimplemented, preadopted, and preferred* technological platform for adolescents.

### Phase 3: Administration

*What components of the selected EBIs should be adapted?* We decided that the adaptation should address the selected core elements of the EBI: (1) ethnic and gender pride role models, (2) sexual health decision making, (3) HIV and STI knowledge, (4) healthy and unhealthy relationships, and (5) negotiating safe sex. It is important to note that several of these activities involve direct, face-to-face, and small group interactions (discussion and simulations) to address situational-partner negotiation strategies and skills, and other activities involve physical skill development and practice (eg, condom application). As these are both essential and not substitutable by texting, it was determined that the adaptation would not be directed toward modification or replacement of the activities associated with the core elements of the workshop. Discussions with experienced health educators noted that each core element and its associated activities supported thematic narratives that recurred in the workshop. Consequently, the core elements of the EBI did not need to be adapted; rather, the narratives that surrounded each core element needed to be extended. Texting, as it turns out, is a powerful, yet efficient, mechanism to accomplish this. Toward that end, all messages would be set in the context of participants in interacting with messages prepared by Tina (one of the health educators).

The adaptation of Afiya involved modifying the telephone scripts and phone-based messages to an SMS text narrative form, aligning with the Afiya core elements. These messages were designed by the developers of Afiya and served as plausible *first approximations* to test the messages, the messaging concepts, and the messaging technology with members of the target population in the formative pilot (phase 8). Thus, the Afiya scripts were predefined narrative structures tailored to the texting form (examples shown in Table 2). As this is a text-based intervention, theater testing was not used; rather, equivalent methods (and supporting theory) designed to examine how humans interact with computers were employed. In general, this is referred to as user experience (UX) analysis and design [88,89].

**Table 2:** Mapping Afiya core elements to texting categories.

Afiya core elements	Primary intervention activities	Associated text category or example
Ethnic and gender pride Role models	Poems and affirmations Media images Role models	*Poems and affirmations (motivational message):* “It is where you are headed not where you are from that will determine where you end up (Marion Wright Edelman)”
Sexual health Decision making	Goal setting Values Sexual health options (condoms, abstinence)	*Sexual health choices (vote poll):* Question: What is ur sexual health choice? Abstinence Condoms Neither Not sexually active Automated responses: Abstinence is the 100% way 2 protect against STDs^a^/HIV. Remember 2 talk with ur partner abt ur sexual health choice Remember 2 use condoms each & every time u have sex. Talk with ur partner abt ur sexual health choice before u have sex Abstinence is 100% STD^a^ protection. Condoms protect u from STD if u use them each&every time. Consider 1 of these options If u decide 2 have sex, condoms protect from STDs if used each & every time. Talk with ur partner abt ur choice
HIV/STD knowledge	Facts about STDs Testing OPRaH Condom do’s and don’ts	*STD testing (cues to action):* Question: When do u need an STD test? Partner has STD Had sex-no condom New partner All of the above Automated responses for (A)-(D): Get tested when new partner, STD symptoms, sex no condom, ur partner has STD. Planned parenthood MTW&F^b^ 8-4:30pm; xxx-xxx-xxxx
Healthy and unhealthy relationships	Personal risk factors Understanding risks What turns you on? Boundary setting Peacefully breaking up	*Boundary setting (cues to action):* Question: Y is it important 2 know ur sexual health boundaries? 2 help stick 2 ur limits 2 communicate them to ur BF A & B Automated responses: One that’s a start! Stick 2 ur limits. Its okay 2 say what ur comfortable doing & what u don’t want 2 do That’s a start! Telling ur partner what ur boundaries r is important so he doesn’t make u uncomfortable or cross the line Exactly! U want 2 b comfortable & happy in ur relationship. Communicating ur boundaries 2 ur bf is healthy
Negotiating safe sex	Healthy communication Sexnarios: negotiation skills Condom excuses and comebacks	*Communication (cues to action):* Question: What type of communicator are u in ur relationship? Aggressive Passive Assertive Automated responses: If u r aggressive, do 1-2 assertive things. Avoid yelling & threatening. B direct & honest. C how he responds. Passive If ur passive, do 1-2 assertive things next time. Avoid the silent treatment. B direct & honest. C how he responds. Great! Continue 2 b assertive when u talk 2 ur partner, even when it gets hard. Be direct & honest. C how he responds

^a^STD: sexually transmitted disease.

^b^MTW&F: Monday, Tuesday, Wednesday, and Friday.

Text messages fit into 1 of 3 categories: motivational messages, vote polls, and cues to action. Motivational messages are one-way messages delivered to participants to serve as inspiration using poetry from Black authors and other leaders. Vote polls are two-way messages initiated by Afiya’s texting platform. The vote polls pose a close-ended question, and participants respond with the letter corresponding to their answer. On the basis of their answers, the system has predesigned responses that will automatically reply to the participant. The automated replies provide information linked back to the Afiya content, serving as a reminder of key messages from Afiya. The cues to action are like vote polls and are two-way messages initiated by the Afiya texting platform. However, cues to action are specifically designed to have automated replies that cue or prompt the participant to engage in a specific action, such as sexually transmitted disease testing or initiating a conversation regarding STI prevention with a sex partner. Each message maps on to at least one of the Afiya core elements presented in the main intervention workshop.

### Phase 4: Production

*How do you adapt and document adaptations?* We analyzed the results of the focus groups and texting and then reviewed them with the HORIZONS and Afiya creators. Initially, the responses to SMS texts were made by a health educator and guided using the modified Afiya phone scripts, and these were adjusted based on the analysis and the consultation with the HORIZONS and Afiya developers. We also initiated the design of computer-assisted self-interview (CASI) questions and instruments as well as focus group–led questions. Measures of fidelity and quality assurance included postsession interviews and participant evaluations. Initial designs of the SMS texting and automated two-way communication were created with help from the Afiya developer with respect to the core elements of the EBI.

In parallel, we began to design the implementation requirements for selecting a commercial texting platform that would best serve our formative texting pilot needs to capture data from a research study, including questionnaires, instruments, and focus group–led questions. At the time of the study, many commercial texting platforms were built for mass marketing. We needed a platform that would allow us to easily set up and automatically send messages based on their cohort or study condition as well as conditional texting responses given a texting question (probe). Additionally, we needed to be able to initially use the platform for recruitment and then transition people into the appropriate study cohort or condition once they consented and were enrolled into the study. Once selected, we would have to code the scripts or texts and conditions as required by the vendor to realize the texting PMI for Afiya.

During this phase, the group drafted an 8-week formative pilot plan to engage the advisory board in the adaptation process. However, given the nature of mHealth technology and uncertainty regarding the specifics of adapting the narratives, we engaged an alternative design method involving an individual texting and response analysis, followed by a group discussion. This resulted in iterations between the administration and production phases, reflecting a development approach often used in software projects to define and refine the UX by tightly integrating interactions and adjustments in design [90].

The overall sequence of the formative pilot is as follows: advisory board members initially participated in a 4-hour HORIZONS workshop (ie, the primary intervention) led by a health educator. Following the workshop, weekly automated SMS text messages were sent that highlighted key messages from the core elements of the workshop for 4 weeks. Advisory board members would activate the weekly text by responding to a weekly text prompt, such as:

Good morning! 2 get ur next HORIZONS convo with Tina, reply to this text with the word WEEK1

They would then receive a text that required a response related to a specific core element covered in the workshop, such as sexual health decision making:

Afiya talked abt sexual health choices: abstinence, condom use or neither. What choice is best for u right now?

**Figure 1:**
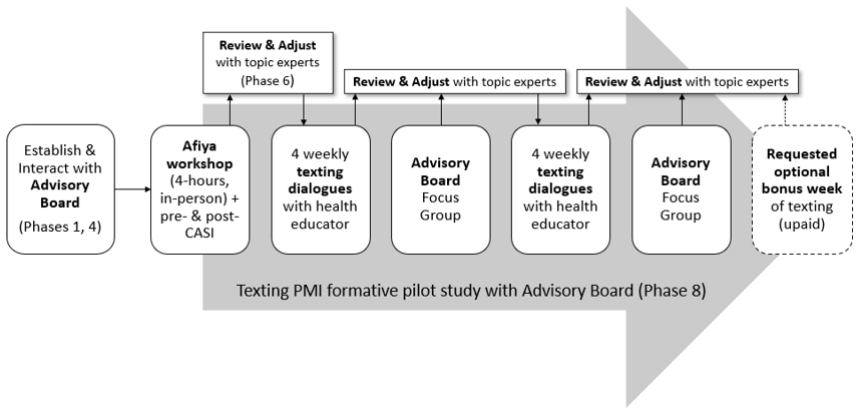
Timeline for formative pilot study. PMI: preventive maintenance intervention.

Subsequent texts were selected using response scripts based on the original phone scripts used in the Afiya phone-based PMI. The timeline for the pilot study is shown in Figure 1. At the end of 4 weeks, the advisory board focus group met to provide feedback. The team reviewed the results and adjusted the scripts and texts. There were then 4 additional weeks of messages followed by the final advisory board focus group, allowing 2 cycles of iterated user response elicitation and refinement (this type of small-scale, iterated focused development is common in UX studies and is similar in theory to spiral development in software engineering but involves rapid testing and turnaround. The presumption is that users may have opinions of their needs and how they would react to a system but after actually engaging a small prototype system the requirements change, which will cause the system to be modified and reassessed in an evolving spiral that converges as the system is developed. This is most common when requirements are ill-defined, as is often found when implementing new types of interactions with technology). Finally, the decisions, designs, and information emerging from phases 1 to 4 were integrated into the first draft, Draft 1.

### Phase 5: Topic Experts

*Who can assist with the adaptation?* Although experts were consulted as needed throughout the process, phase 5 involved additional experts whose input was specifically necessary to help review the adapted intervention design (content and form). In this case, additional experts were solicited: (1) experts in designing CASI items and psychosocial instruments relevant to the purpose and population, (2) representatives from selected third-party texting companies to determine capabilities, constraints, and pricing, and (3) those who had specific Health Insurance Portability and Accountability Act knowledge regarding ethical-legal implications of the possible SMS texting content and data privacy or protection issues. We generated initial estimates of total program costs and had them reviewed by the team and administrators. Primary cost estimates included the texting company, a graduate research assistant (GRA), and participant incentives.

### Phase 6: Integration

*What is going to be included in the adapted EBI?* Phase 6 involved the integration of the results from Draft 1 (phase 4) with the results of the topic experts’ review of the fidelity of the script or message designs relative to the original Afiya intervention core components (phase 5) to create Draft 2. Project members reviewed the pre- and post-Afiya workshop CASI measures (the workshop was offered when phase 4 CASI materials were completed and vetted) and revised the instruments to (1) reduce their length and (2) include assessment items appropriate for evaluating an SMS-based intervention. We finalized the modified Afiya scripts for a texting algorithm that would be used by the health educator in the postworkshop texting in phase 8. These were reviewed by Afiya developers and the advisory board. Our team produced Draft 3 after completing the workshop and after text communication with advisory board members in phase 6.

### Phase 7: Training

*Who needs to be trained?* Once the decisions were made regarding the adapted text intervention (scripts, SMS texts, texting platform), intervention-specific training materials were developed for the formative pilot. Health educators were trained on how to implement the Afiya workshop; materials were already available, with minor modifications introducing SMS texting and approved by the Afiya developers. Health educators were trained on the SMS text scripting component and texting platform use and were part of the development process. One of the study principal investigators and a GRA were trained to develop and manage the texting platform and messages. Data analysts were trained on the texting platform’s data components such that texts and associated data could be acquired from the company.

### Phase 8: Testing

*Was the adaptation successful?* The definition of success and the form of the testing depend on the context of the adaptation, but the general objectives remain. As our adaptation did not include delivery to a different target population, we modified the two-step testing processes of phase 8.

In Step 1, we assessed the *adaptation efficacy* by conducting an 8-week formative pilot study with the advisory board members, as shown in Figure 1. This important validation step addresses whether the intervention was effectively adapted and implemented correctly, involving close monitoring and specific types of data acquisition and feedback and testing all the operational components including health educator training and performance of the texting company. We decided against a *pure* formative pilot (ie, engaging a new set of participants) because of the extensive involvement of the advisory board and HORIZONS and Afiya developers, the retention of the same target population as Afiya, and the success of the iterative design process with the advisory board for the formative pilot. This approach was extremely helpful and informative in designing the final product of this stage, the randomized controlled trial (RCT) efficacy study.

Step 2 involves assessing the *short-term intervention efficacy* of the PMI by conducting an *RCT feasibility study* to test the efficacy of the PMI over an extended duration with the target population. Regarding Part 2, a 3-arm RCT efficacy study (standard of care, Afiya workshop only, and Afiya+texting) was designed. This has been approved by Emory’s Institutional Review Board and is being initiated.

## Results

As noted, the formative study involved the advisory board members (n=6) actively participating in adapting Afiya to a texting platform, and they were compensated for their time and effort. Focus groups with the advisory board were conducted midway through the pretest and after the last week, allowing 2 cycles of iterated user response elicitation and refinement. Examples of the feedback after week 4 are summarized in Textbox 1. For this focus group, we sought to distinctly examine 4 categories of texting issues: timing, wording, content, and form. The narrative analysis was very useful in extracting issues regarding texting characteristics, such as timing (eg, *thank you* messages came too early in the texting series), wording (eg, questions were judged as being clear and simple), content (eg, inspirational messages were appreciated), and form (eg, desired more individualized feedback). Getting the text wording *correct* for the target audience was also a continuous improvement process to facilitate culturally appropriate narrative engagement via text. Frequent use of commonly agreed-upon abbreviation dialects in texting results in these abbreviations being rapidly absorbed into the language and automatically (linguistically) recognized as *words* [91], reflecting how humans socially adapt to a technology (and its constraints). On the basis of the first focus group with the advisory board, the following modifications were made for the second 4-week session:

1. We added a motivational or inspirational message each week in addition to the regular text, such as “It is where you are headed not where you are from that will determine where you end up” (Marion Wright Edelman).
2. We added more complex response scripts to the prompts because participants felt some of the texts were too open ended and not specific to them.
3. We provided individualized feedback and advice during a time for open questions about any topic.
4. We gave advisory board members a specific window of time to chat with the health educator
  live
  instead of always getting the automated message responses.

**Results from the advisory board texting formative pilot (texting issues and examples of reactions; weeks 1-4).**

Timing:

- Texting frequency should be increased; daily texts are acceptable
- No preferences for weekday vs weekend texts
- Texts should not come too early in the day; better to get texts starting mid-day and continue through early evening
- Participants indicated that the Thank You message came too early in the text series and would have preferred a more in-depth conversation via text

Wording:

- Text wording was straight-forward and participants liked that the messages were not *sugar coated*
- Questions were clear and simple

Content:

- Texts about sexually transmitted diseases were favorite and made them think about their behaviors
- When discussing relationships, participants emphasized that content should focus on communication in relationships and engaging in safe behaviors. They also wanted the discussion to expand to other facets of relationships (eg, quality of relationship, satisfaction with relationship)
- For more complex issues like relationships and communication, the participants requested more feedback and information to help keep them on track
- Participants indicated that any topic would be acceptable by text if they knew where their answers were going and who was reading or responding to the texts
- Wanted a motivational or inspirational message or quote each week

Form:

- Dissatisfied that there was not a person live on the other end of the texting
- Disliked texting with a computer that could not be more interactive and have a conversation with a live person
- Wanted more individualized feedback and advice to come with the texting program

All advisory board members participated during weeks 5 and 6; participation decreased during the second half of the pilot. Reasons for nonparticipation were as follows: (1) phone temporarily disconnected and (2) busy during the specific designated conversation window (the scheduled time to interact). Within the final focus group, comments were sought regarding their overall experience and thoughts for the PMI. Examples of the feedback after week 8 are summarized in Textbox 2. These fell into 4 primary categories: HIV and STI information, context-specific social network maintenance, social engagement, and other ecologically relevant information or content. Participants sought additional information on related programs and topics beyond those addressed in the workshop. They also reported that the social connection with the health educator was indeed maintained, facilitating engagement. An interesting consequence of the pilot was the value of the social network that emerged within the context of the pilot and the role that delivery platform could play in maintaining that social network.

**Results from the advisory board formative pilot (weeks 5-8, final focus group).**

HIV and sexually transmitted infection information:

- Participants wanted to make sure that information about new programs they may be able to participate in would be sent to them
- Participants requested that the texting program also be a forum to let participants know about group meetings and group events surrounding related topics, such as medical or health service options (clinics that provide free or reduced-price services), education events, and training activities

Context-specific social network maintenance:

- They stated that they enjoyed the texting but loved the groups
- They emphasized repeatedly a desire to continue meeting (in person) with the groups
- They expressed a design to have a forum to stay in touch in case their phone numbers change
- Participants noted that they liked having greater time to talk and the ability to have more detailed, back-and-forth conversations

Other ecologically relevant information:

- Participants stated they wanted information on the following topics: relevant community events, parenting, housing (different locations, pricing, etc), job information (job fairs, career info, etc), and health insurance information

Social engagement:

- Participants stated that they felt like they *were texting with a friend* and felt very comfortable texting with the health educator. Important characteristics for health educator: woman, relatable, trustworthy, the same person over time to develop a relationship with, knowledgeable person who can provide accurate information

Postadaptation, we analyzed how advisory board members engaged during the adaptation process, focusing on the emergent themes of interactions, to define an initial set of personas. Personas are specific, but hypothetical, individuals possessing core sets of relevant characteristics (eg, situations, beliefs, goals) that differentially influence the interaction narratives. The persona analysis defined 5 personas and linked each persona to counseling scripts from the Afiya intervention that reflected the context of prevention needs based on the situational narrative, the condom use behavior, and the current status of any relationships. Persona results were reviewed and revised by Afiya health educators and the project team. Note that personas are usually stable (as a descriptive model for the target population), but individuals may move between personas. If certain characteristics change, persona classifications can be modified (eg, a *Shannon* may turn into a *Lexie*). The results are shown in Table 3.

**Table 3:** Results of the persona analysis for the Afiya texting adaptation.

Characteristics	Shannon	Tonya	Kia	Shauna	Lexie
Narrative	Shannon has been in a long-term relationship with her boyfriend for almost a year. They stopped using condoms after the first couple of months	Tonya is single. She just got out of a relationship and needs some time to focus on herself and her schoolwork	Kia is dating someone, but also *talking* to a couple of other guys. She does not have a formal boyfriend but has a consistent sex partner. However, they are not in an exclusive, monogamous relationship	Shauna is starting a new relationship with a guy. They’ve been seeing each other for a month. She is abstinent and is ready to let him know her sexual health choice	Lexie has both a boyfriend and a casual sex partner
Condom use	Shannon has discontinued the use of condoms	Tonya is not having sex right now but wants to use condoms when she does get into a relationship again	Kia uses condoms with her sex partners	Shauna does not need condoms at this time as she is not having sex	Lexie uses condoms with her casual partner but not with her boyfriend
Relationship status	Shannon has had a boyfriend for 1 year	Tonya has no boyfriend	Kia has no boyfriend but has multiple partners	Shauna has a new partner	Lexie has concurrent sex partners
Link to Afiya intervention scripts	Script D: no to condom use	Script A: no boyfriend; me time	Script C: yes to condom use	Script B: yes to abstaining	Script E: inconsistent condom use

Finally, upon request from the advisory board members, we provided them with the opportunity to text in with any questions they may have. This was an opportunity for them to seek additional information that they may not have learned through the program thus far. The text lines were open for 7 days. advisory board members were not compensated for texting during the bonus week 9; it was an opportunity for them to get any additional information from a health educator before the program ended. Half of the advisory board members participated in the unpaid bonus week of texts. The chat line parameters were as follows:

1. Chat lines open for 7 days.
2. You will not get paid for these texts.
3. You can ask about anything you have questions or concerns about.
4. You will get a response from Tina within 24 hours.
5. If you have an emergency, you should always call 911.

## Discussion

The motivation for this paper was provided by the significant disparities in STI and HIV health risks among African American youth. Ethnic minorities in the United States have STIs 30 times greater than that in White, middle-class populations [92]. To address this disparity, coupled with scarce funding and staffing resources, new forms of digital technology solutions are constantly being sought, proposed, and attempted to curb the STI and HIV risk behaviors of adolescents and young adults, who are at most risk [67]. However, few guidelines currently exist to inform designers how digital media can be used in ways that can impact the efficacy of an intervention.

This paper describes a conservative approach to contribute to this effort. Specifically, this paper describes the development of a systematic process to incorporate simple texting messages as an adjunct program maintenance intervention to an evidence-based sexual health intervention for African American adolescent women.

The systematic approach of the ADAPT-ITT framework was clear and effective in phases 1 through 7. The reason was the reliance on the well-proven and understood primary intervention (HORIZONS) and the prior efforts (and developers) experienced in adapting that intervention to a communication-based PMI (Afiya). However, in developing technological implementations, we found it necessary to engage a sample of the target population with the PMIs directly, rather than the theater-testing approach suggested in phase 1.

The type of small-scale, iterated development approach with the formative pilot is common in UX design and software engineering. The presumption is that users may have *beliefs* about their needs and how they would react to such a system, but after *engaging* a system, many of their beliefs and preferences (therefore the requirements) change. This is most common when implementing new (to users) types of interactions with technology. Consequently, the formative pilot was conducted in phase 8: Testing, as it required a full implementation of the PMI to gather efficacy and implementation data.

As shown in Figure 1, the formative pilot included 2 focus group sessions interspersed within the SMS texting intervention. This allowed intermediate analysis of the 4-week experience to be reviewed by the developers (and any relevant topic experts) and adjust the SMS texting narrative characteristics. Thus, the formative pilot testing in phase 8 *looped back* to elements of phases 4 to 7.

It is unlikely that technology will elevate an ineffective intervention to one that is effective—automating a weak process generally results in an automated weak process. There is also a risk that poorly designed and implemented technological components may lower the efficacy of a proven intervention. The selection of texting was seen as a key technology commonly adopted by this group as a mechanism for sociocultural connectivity, thus having the potential to increase the uptake of extant interventions by extending the narratives that began in the core EBI workshop activities.

Early and repeated inclusion of the target population, advisory board, was essential in the development. In particular, the generation of personas from the advisory board participants provided insight into important narrative variations for subsequent delivery design. Key considerations for end users should be sought early in the process of app (or other digital) behavioral intervention design to ensure both short- and long-term engagement [93-95]. In the paper by Pettifor et al [13], titled Adolescent Lives Matter, they argue that “adolescents should be involved throughout the process from design to implementation.” This is supported by a recent systematic review of mHealth apps for adolescent users and concluded that there is a fundamental difference between adult and adolescent preferences in apps, where apps for adolescents need features that support decision making [91]. In summary, a quote by Glasgow et al [96] echoes the spirit of this paper’s intent:

Controlling the epidemic will not only require bringing successful interventions to scale but also tailoring them to vulnerable and marginalized populations and understanding the social, cultural, and institutional contexts in which interventions are delivered.

Page S26

In summary, 4 general suggestions can be made regarding how to proceed when considering mHealth solutions.

### Suggestion 1

*Do not start with the technology. Start with the EBI-based workshop and then discern how to adapt both.*

EBIs (and the underlying theoretical base) define core elements that have demonstrated effective results. Technology alone cannot *increase efficacy to significance* but has the potential to decrease efficacy to insignificance. This leads to the necessary inclusion of methods that address user-centered issues when designing the technological components.

### Suggestion 2

*Technological adaptations of EBIs need to explicitly address the user’s experience as developers design, evaluate, and implement the technical components of an intervention.*

Participants of technology-based intervention components are indeed *users* interacting with a service. Much is known about how to design, evaluate, and implement user services in general and how to assess the UX with technology in particular. This leads to an important conclusion regarding how to view the UX with adapting an EBI using a texting PMI.

### Suggestion 3

*Texting is less of a technical choice and more of a social choice.*

Texting is not about creating a technological intervention and then trying to get individuals to adopt and to disseminate it. Everyone who has a mobile phone has the application; most people know how to use that application; most people have adopted that application for use. There is another element to consider; people use it for the same reason: *engaging in narratives with others*. Thus, a critical communication pathway already exists but it is limited. Interventions that use mHealth and texting compete for the user’s attention and the user’s time. Regarding the EBI, attention is gained, for example, via the EBI workshop (as demonstrated for this population for this health problem).

However, the narratives occurring in the workshop must be maintained through the initial (and continuing) establishment of *trust*, which mediates between information quality and use [97]. All health educators know this. By understanding the cultural components of the target population’s preferences, choices of how the technology is used can engender that trust, thus sustaining critical narrative connections. Indeed, today’s young adults value technology as *a way to enhance, not replace* their interactions with their health providers [37]. This leads to our fourth suggestion.

### Suggestion 4

*Texting as a PMI used with an EBI workshop is less about reminding and more about re-engaging narratives from the core intervention.*

Texting can be used to extend the information and skills covered in an initial workshop or session. The Afiya intervention did not cover new information via texting. All information was presented in the in-person initial workshop. Therefore, testing then served to boost and further highlight details covered in the initial intervention. For example, the initial Afiya workshop demonstrated communication and condom use skills. The follow-up texts used terminology from the original intervention to remind or cue participants to the content. Subsequent text messages would then ask the participant about her communication style or whether she talked with her partner about her sexual health choice. The use of SMS in behavioral research should consider having an initial in-person presentation of information and using SMS to serve as a reminder of the information, resources, and skills presented.

### Limitations

The challenge in future research will be to transition from administration on a small scale to a larger scale while maintaining the tailoring and personalization participants preferred in this study phase. Additional study challenges included intervention length. This study included 8 to 9 weeks of two-way texting between a health educator and participants. To allow SMS to serve as a true intervention booster and to increase the maintenance of study outcomes, the intervention length may need to be implemented over a longer period. We also discovered that there is one more critical persona: the health educator; that is, each participant develops a mental model of the individual with whom she would be texting. Therefore, one goal moving forward is to ensure that the health educator (for a workshop) conveys a consistent persona in future iterations of the study.

Finally, and admittedly, there is a lack of strong economic data to support the use of mHealth behavioral interventions [98,99], and this pilot study did not examine the economic implications. We will be engaging in economic evaluations in our PMI RCT feasibility pilot study (step 2 of phase 8, Table 1). We believe that the systematic design and development of mHealth solutions based on strong evidence-based foundations can begin to show economic value as critical adjuncts (PMIs) by extending the efficacy of interventions. This requires systematically determining how a *technological form can be adapted to an evidence-based intervention.* However, this is both ill-defined and underresearched, leaving distinct gaps in the design, implementation, and acceptance of mHealth apps [100,101]. The incorporation of mHealth technologies necessitates considerations of how the current theories and practices employed can accommodate these technologies [102] and argues for the inclusion of theories and practices from other disciplines [103,104], especially those disciplines associated with explicitly studying how humans use technology [105,106]. This paper offers an approach to address such gaps.

### Conclusions

In conclusion, given that digital technology is seen as an important component of addressing the STI and HIV epidemic in African American adolescents [67,107] as well as for public health innovation in general [108], we demonstrated how to systematically adapt an existing HIV and STI EBI designed for telephone-based health educator communication with African American females to one that is based on SMS texting. The ADAPT-ITT approach was used to navigate the adapted intervention. As recommended, we involved the target user group, sexual health experts, behavior change experts, software developers (SMS companies in this case), and research experts [109], a considerable strength of the study methodology. It is important to note that if mHealth technologies are to be components of an intervention, it is inappropriate to assume (explicitly or implicitly) that *technology is neutral*. This paper represents an initial step toward engaging a more systematic process, ADAPT-ITT, that will provide guidance for EBIs to extend the duration (and retain the integrity) of their impact using the most common communication technology on earth—texting.

## Abbreviations

ADAPT-ITT: assessment, decision, adaptation, production, topical experts-integration, training, testing
CASI: computer-assisted self-interview
CDC: Centers for Disease Control and Prevention
EBI: evidence-based intervention
GRA: graduate research assistant
mHealth: mobile health
PMI: preventive maintenance intervention
RCT: randomized controlled trial
STI: sexually transmitted infection
TGP: theory of gender power
UX: user experience
